# Late-Onset 22q11.2 Deletion Syndrome With Mild Cardiac Phenotype: A Unique Adult Presentation Diagnosed at 45 Years of Age

**DOI:** 10.7759/cureus.50367

**Published:** 2023-12-11

**Authors:** Anasofia Elizondo-Plazas, Graciela Areli Lopez-Uriarte, Jose Gerardo Gonzalez-Gonzalez, Rene Rodriguez-Gutierrez, Laura Martinez-Villarreal, Angel Sebastian Trevino-Juarez, Camilo Daniel Gonzalez-Velazquez

**Affiliations:** 1 Department of Medical Genetics, University Hospital “Dr. Jose Eleuterio Gonzalez” Universidad Autónoma de Nuevo León, Monterrey, MEX; 2 Endocrinology Division, Department of Internal Medicine, University Hospital “Dr. Jose Eleuterio Gonzalez” Universidad Autónoma de Nuevo León, Monterrey, MEX

**Keywords:** heart septal defects, hypocalcemia, hypoparathyroidism, digeorge syndrome, 22q11 deletion syndrome

## Abstract

This case report presents a detailed exploration of an adult-onset 22q11 deletion syndrome, a rare genetic disorder typically diagnosed in children. The report highlights the diagnostic challenges posed by this atypical presentation, emphasizing the need for clinicians to consider such conditions in differential diagnoses, especially in adults. This case is remarkable for its late onset and mild symptoms, which significantly deviated from the common pediatric presentation, including hypocalcemia due to hypoparathyroidism and a fenestrated atrial septal defect without significant hemodynamic implications. The importance of recognizing the broad phenotypic variability of the syndrome and the implications for clinical practice are discussed, providing insights into the genetic and phenotypic diversity of the condition. In conclusion, this case illuminates the diverse clinical spectrum of adult-onset 22q11 deletion syndrome, emphasizing its relevance to clinical practice.

## Introduction

22q11.2 deletion syndrome is recognized as the most frequent interstitial microdeletion in the human population, with an estimated incidence of 1 in 2,000 live births [1]. Typically, this condition manifests sporadically, accounting for about 90% of cases, while the rest follow an autosomal dominant inheritance pattern [2]. Traditionally, clinical diagnosis has been centered on neonates who present with a triad of symptoms: hypocalcemia, immunodeficiency, and conotruncal cardiac defects [3]. The pathogenesis of the syndrome involves the loss of essential genetic material within the 22q11.2 region, crucial for normal embryological development [4]. This loss leads to aberrant morphogenesis, particularly affecting the derivatives of the third and fourth pharyngeal arches, resulting in the hallmark features of facial dysmorphisms, cardiovascular malformations, and hypocalcemia in newborns, which typically prompt clinical suspicion [3,4]. Notwithstanding, the syndrome exhibits a broad spectrum of phenotypic variability.

This case report describes a rare instance of 22q11.2 deletion syndrome in a 45-year-old female, marked by adult-onset hypocalcemia and a mild, atypical presentation of symptoms. Unlike typical pediatric cases, the diagnosis was delayed due to subtle clinical signs, including a minor atrial septal defect identified through echocardiography. This atypical adult presentation emphasizes the importance of heightened clinician vigilance regarding 22q11.2 deletion syndrome in adults, underscoring its genetic and phenotypic diversity and the risk of underdiagnosis in older patients. This case report aims to contribute valuable insights to the medical literature by emphasizing the rarity of 22q11.2 deletion syndrome in adults, advocating for consideration across diverse age groups, and highlighting the imperative for increased clinical recognition regarding its genetic and phenotypic variability.

## Case presentation

**Figure 1:**
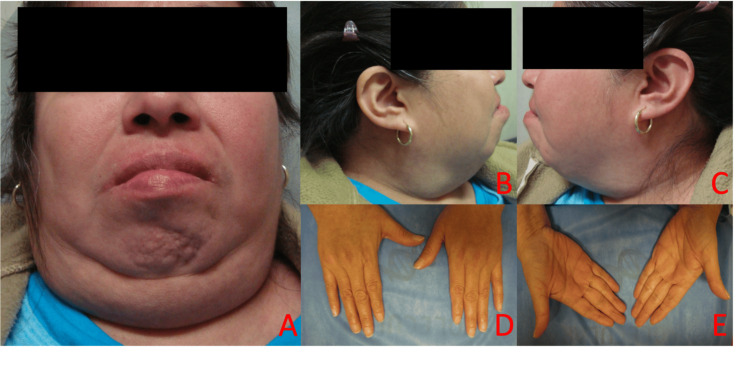
Multimodal facial and hand phenotypic characterization. (A) Frontal facial view. (B and C) Lateral facial views. (D) Dorsal view of hands. (E) Palmar view of hands.

A 45-year-old woman with a history of primary Hashimoto’s thyroiditis presented with myotonic seizures in the emergency department. Notably, she was the firstborn of a non-consanguineous, ostensibly healthy couple and had a documented history of underperformance in school without further cognitive deficits. Upon evaluation, her vital signs were stable, and she was alert and oriented. The physical examination revealed a soft, non-tender goiter, alongside features such as short stature, obesity (body mass index: 31.6 kg/m^2^), and distinctive facial characteristics (Figure 1).

Laboratory findings were significant for severe hypocalcemia and hyperphosphatemia, which prompted immediate intravenous calcium supplementation. This intervention was initiated before the assessment of parathormone levels, despite the clinical suspicion of hypoparathyroidism.

As her hospital stay progressed, a consultation with Clinical Genetics was requested to explore the potential of a polyendocrine syndrome, taking into account her physical features and laboratory results. The genetics evaluation corroborated facial dysmorphism; her head circumference fell below the third percentile, characterized by straight palpebral fissures and semi-arched eyebrows. Additionally, a thin nasal bridge, bulbous nasal tip, and low-set ears with underdeveloped helices and fused lobes were documented. The patient’s speech, although nasal, was intelligible, and her behavior was marked by a pronounced dependency on her parents and unprovoked episodes of crying. These clinical observations led us to suspect a microdeletion syndrome. Subsequent microarray-based comparative genomic hybridization analysis unveiled a 2.5 Mb deletion in the cytogenetic region of 22q11.21, confirming a diagnosis of 22q11.2 deletion syndrome. The deletion impacted 75 genes, including *DGCR6*, *COMPT*, and *ZNF74*, which are linked to psychiatric conditions, and *TBX1*, which plays a crucial role in cardiac embryogenesis. Remarkably, despite the haploinsufficiency typically producing serious conotruncal congenital heart defects, the patient’s mild cardiac phenotype had not manifested clinically throughout her life. The transthoracic echocardiogram revealed only slight dilation of the right cardiac chambers and Doppler flow indicative of a fenestrated atrial septal defect, without any resultant shunt or hemodynamic implications.

At present, the patient is under continued care, maintaining a clinical euthyroid state (thyroid-stimulating hormone: 3.2 μIU/mL) and showing no hypocalcemic symptoms with her current regimen of calcium carbonate 600 mg orally with meals and calcitriol 0.25 µg daily. She has exhibited no signs of heart failure nor audible murmurs that could be attributed to the atrial septal defect identified.

## Discussion

In contemporary medical practice, the emphasis on diagnostic testing may inadvertently narrow the scope of clinical assessment for trainee physicians, potentially obscuring the broader clinical context and leading to missed syndromic diagnoses. This trend could notably delay the identification of atypical presentations of 22q11.2 deletion syndrome (22q11DS), resulting in prolonged hospitalizations and diminished patient quality of life. As illustrated in this case report, atypical adult-onset presentations of 22q11DS may not exhibit classical symptoms, emphasizing the importance of a broader diagnostic perspective. The absence of pathognomonic indicators in such cases can challenge clinicians’ abilities to make timely diagnoses. Therefore, emphasizing the need for trainee physicians to consider rare syndromes in adult populations, even in the absence of typical pediatric presentations, is vital in improving diagnostic accuracy and patient outcomes.

Congenital heart disease (CHD) manifests in 75-80% of individuals with 22q11DS, with conotruncal defects being most prevalent [5]. For instance, individuals with subtle cardiac abnormalities or isolated non-cardiac symptoms may not immediately raise suspicions of 22q11DS, resulting in diagnostic delays. Such cases underscore the importance of recognizing the syndrome’s variable clinical patterns and the potential pitfalls in relying solely on laboratory tests for diagnosis. While these cardiac abnormalities were historically deemed fatal during childhood, recent data suggests that individuals with major CHD now have a survival probability of about 72% to the age of 45, and up to 95% for those with milder forms [6]. With the advent of sophisticated molecular cytogenetic diagnostics, clinical suspicions can be validated, allowing for tailored treatment strategies. However, the literature on this patient demographic is limited, predominantly comprising case reports and a handful of small cohort studies. The increasing life expectancy of these patients presents a new set of challenges, particularly in adult manifestations of the syndrome, which necessitates precise genetic counseling and enhanced care.

The diverse clinical manifestations of 22q11DS necessitate a recognition that there is no singular pathognomonic indicator for the condition. Therefore, despite traditionally being viewed through a pediatric lens, clinicians should be vigilant for cues indicative of this syndrome in adults. It is essential for medical trainees to become adept at discerning the varied clinical patterns associated with 22q11DS to provide comprehensive care. A reliance on laboratory tests may divert focus from the underlying etiology to symptomatic lab values, which could lead to suboptimal management. A holistic approach, valuing the insights gained from a meticulous anamnesis and physical examination, is paramount in fostering a complete understanding of the patient’s condition. Implementing systematic screening protocols or raising awareness among healthcare providers about the condition’s diverse manifestations in adults may lead to earlier diagnoses and improved patient outcomes.

## Conclusions

This case report underscores the importance of vigilance and comprehensive evaluation in diagnosing genetic conditions such as 22q11.2 deletion syndrome in adult populations. It challenges conventional clinical expectations and illustrates the necessity for clinicians to remain alert to the possibility of atypical presentations, especially in genetic disorders traditionally associated with pediatric patients. The report serves as a crucial reminder of the genetic and phenotypic diversity inherent in conditions such as 22q11 deletion syndrome and highlights the importance of considering such diagnoses in adult patients presenting with unusual symptoms.
